# Use of cultivated plants and non-plant remedies for human and animal home-medication in Liubań district, Belarus

**DOI:** 10.1186/s13002-017-0183-6

**Published:** 2017-10-03

**Authors:** Renata Sõukand, Yanina Hrynevich, Julia Prakofjewa, Tatsiana Valodzina, Iryna Vasilyeva, Jury Paciupa, Aliaksandra Shrubok, Aliaksei Hlushko, Yana Knureva, Yulia Litvinava, Siarhei Vyskvarka, Hanna Silivonchyk, Alena Paulava, Mare Kõiva, Raivo Kalle

**Affiliations:** 1 Department of Environmental Sciences, Informatics and Statistics, Università Ca’ Foscari Venezia, via Torino 155, 30172 Mestre, VE Italy; 2 Estonian Literary Museum, Vanemuise 42, 51003 Tartu, Estonia; 3 The Center for Belarusian Culture, Language and Literature Research, Surhanava St., 1, Bldg. 2, 220072 Minsk, Belarus; 4 Valozhynski district, v. Vialikaya Dajnava, Padhornaya st., 118, 222352 Minsk Region, Belarus; 5 Liubań District Culture Center, Pershamajskaya st., 30, 223820 Liubań, Belarus; 6 The Belarusian State University of Culture and Arts, Rabkorauskaya st. 17, 220007 Minsk, Belarus; 7 Estonian University of Life Sciences, Institute of Agricultural and Environmental Sciences, Kreutzwaldi 5, 51014 Tartu, Estonia

**Keywords:** Belarus, Non-plant remedies, Cultivated plants, Wild plants, Medicinal plants, Local knowledge, Ethnobotany, Ethnopharmacology, Ethnoveterinary, Liubań

## Abstract

**Background:**

To use any domestic remedy, specific knowledge and skills are required. Simple logic dictates that the use of wild plants in the context of limited interaction with nature requires prior identification, while in the case of non-plant remedies and cultivated plants this step can be omitted. This paper aims to document the current and past uses of non-plant remedies and cultivated plants in the study region for human/animal medication; to analyze the human medicinal and veterinary use areas in the context of the remedy groups; to qualitatively compare the results with relevant historical publications; and to compare the intensity and purpose of use between the remedy groups.

**Methods:**

During field studies 134 semi-structured interviews were conducted with locals from 11 villages in the Liubań district of Belarus. Currently used home-remedies as well as those used in the past were documented by employing the folk history method. The subject was approached through health-related uses, not by way of remedies. Interview records were digitalized and structured in Detailed Use Records in order to ascertain local perceptions. An Informant Consensus Factor (FIC) was calculated for remedy groups as well as for different use categories.

**Results:**

In the human medication area the use of nearby remedies was neither very diverse nor numerous: 266 DUR for 45 taxa belonging to 27 families were recorded for cultivated plants along with 188 DUR for 58 different non-plant remedies. The FIC values for both remedy groups were lower than for wild plants. In the ethnoveterinary medicine use area there were 48 DUR referring to the use of 14 cultivated plant taxa from 12 families and 72 DUR referring to the use of 31 non-plant remedies. The FIC value for the whole veterinary use area of cultivated plants was relatively low, yet similar to the FIC of wild plants.

**Conclusions:**

Differences between remedy groups were pronounced, indicating that in domestic human medicine cultivated plants and non-plant remedies are either remarkably less important than wild ones or not considered worth talking about. In ethnoveterinary medicine non-plant remedies are almost equally important as wild plants, while cultivated plants are the least used. People in study area seem to still more often rely on, or are more willing to talk to strangers about, wild plants, as promoted by both official medicine and popular literature.

## Background

Academic human and veterinary medicine has advanced rapidly during the last few centuries. Educated help has become very effective, widespread, and rather affordable (or even free) in most European countries, making academic medicine the first and often only choice for the majority of Europeans. Yet, complementary and alternative medicine remains popular and is used for treating specific alignments [1]. Herbal medicines are often highlighted as the most popular among different treatments (for example [2]).

Quite logically, most researched remedies in the enthobotanical literature appear to be plants, leaving aside other possible means used for healing in the home setting. Research on the traditional or local medicinal (and very rarely veterinary) use of plants is quite often paired with the documentation of the food-use of (wild) plants [3, 4], while non-plant remedies are rarely documented, and if they are they concentrate on only a single remedy (such as [5]). Yet, recent ethnobotanical research has shown that in Eastern Europe a wide variety of non-plant remedies are still used for healing different human and (more rarely) animal health conditions [6].

In order to use any domestic remedy, specific knowledge and skills regarding what remedy to use to treat a specific disease and how to make it, as well as how to apply or preserve it, are required. For wild plants that grow outside of direct reach, an additional set of specific knowledge is needed, including where they can be found and how to recognize or differentiate them from similar taxa. Hence, the use of non-plant remedies requires a less detailed knowledge set compared to the one needed for the use of plants.

Cultivated plants fall somewhere in between wild plants and non-plant remedies in terms of availablity at home: they grow in close proximity to people, and hence are more or less at hand, yet one has to possess specific knowledge regarding their cultivation. Moreover, through cultivation the taxa are well known, and as a result the identificaton step needed for wild taxa can be omitted. A study examining changes in medicinal plant use in Estonia over the course of a century suggested that the use of plants tended by humans has increased over time as people have fewer encounters with nature and they feel more confident using plants they know for certain [7]. This suggests that the use of cultivated plants could also now be preferred or at least as popular as the use of wild ones, as the historical legacy is quite similar to that in Estonia. Yet, in Belarus, no specific studies of medicinal plants cultivated in home gardens have been conducted. However, some additional data apart from a list of the most common cultivated plants can be found in the ethnographic literature. For example, the most diverse collection of archival data from the Polish-Lithuanian-Belarusian borderland was collected in the 1930s by a Polish ethnographer from Lviv, Adam Fischer. These data were originally gathered for publication in the first part of the Lexicon of Slavic beliefs and customs, dedicated to plant uses in traditional Slavonic culture, but this objective has not yet been realized. Modern Polish researchers have analyzed and published some archival data [8]. In Fischer’s archival data home garden plants are represented by 18, mainly ornamental, species: peony (*Peonia* sp.), marigold (*Calendula officinalis*), *Tagetes* spp., mallow (*Malva* sp.), garden nasturtium (*Tropaeolum majus*), perennial phlox (*Phlox paniculata*), panicled aster (*Symphyotrichum lanceolatum*) and sneezewort (*Achillea ptarmica*). Two species were mentioned as potted plants, namely agave (*Agave* sp.) and surprisingly dwarf everlast (*Helichrysum arenarium*). Gourd (*Lagenaria siceraria*) was planted for its fruits, which then were used indoors – mounted on wardrobes and tile stoves for ornamental purposes. This group comprises valuable and useful plants which could simultaneously be treated as ornamental and medicinal: mint (*Mentha* spp.), rue (*Ruta graveolens*), poppy (*Papaver* sp.), elecampane (*Inula helenium*) and southernwood (*Artemisia abrotanum*) [8].

In the post-war period since the 1960s the use of cultivated medicinal plants has become the subject of study in pharmacology as well as agricultural and veterinary sciences, for example ([9, 10], etc). In the 1990s and 2000s, brochures and encyclopedias on the use of cultivated plants from home gardens for medicinal purposes gained in popularity, for instance ([11, 12], etc).

During ethnobotanical and ethnomedicinal fieldwork conducted in the Liubań (Любань) district of Belarus we documented, along with the use of wild plants (outlined in [13]), the use of all non-plant remedies pertaining to humans and animals as well as the medicinal and veterinary use of cultivated plants. Initial analysis of the results revealed that the uses of both remedy groups are quite limited, in both nomenclature and diversity of use, compared to wild food plants. However, the number of interviewees and depth of the interviews allow for deeper insight. Moreover, as the historical literature on Belarus covers non-plant remedies to a greater extent than the use of medicinal plants, it is a good opportunity to determine if recorded historical uses of non-plant remedies are still practiced or at least remembered.

As similar studies rarely assess the use of non-plant remedies, we decided to examine the subject and compare the intensity and diversity of use of different kinds of remedy groups – in this case non-plant remedies as well as cultivated and wild plants (the last group was covered in [13]) – to establish if there are any differences in use-patterns within or between the groups. With this information we aim to establish if virtual proximity to the domestic arena and diverse practical availability influence the importance of a remedy in domestic human and veterinary medicine.

The specific aims of this study are: 1) to document the current and past uses of non-plant remedies and cultivated plants in the Liubań district of Belarus for human/animal medication; 2) to analyze the human medicinal and veterinary use areas in the context of non-plant remedies and cultivated plants; 3) to qualitatively compare the results with relevant historical publications regarding non-plant remedies and cultivated plants in Belarus; and 4) to compare the intensity and purpose of use between three remedy groups: non-plant, cultivated plants and wild plants.

## Data and methods

### Subjects of analysis

To examine the idea of the importance of the perceptional distance of primary ingredients to the domestic arena, two nearby remedy groups were thoroughly assessed:

- **Cultivated plants**: this encompassed all plants that were intentionally cultivated in order to be eaten (at least some part of them), even if they run wild (like many Lamiaceae species) or are native and grow wild, but are, for medicinal reasons, collected mainly from cultivated specimens (like two *Ribes* species). This group also included self-planted tobaco (*Nicotiana* spp.), ﻿bedding plants (like *Paeonia* spp.), potted plants (like *Pelargonium* spp. or *Callisia fragrans*), and plants obtained from outside the household (store-bought) and clearly of foreign origin (*Piper nigrum, Camellia sinensis*). The main criteria for identifying plant as “cultivated” was that it was either grown by the person themself or obtained in a “ready to use form” so that no special identification was needed.
- **Non-plant remedies**: this comprised those parts of plants not permitting or not requiring, from the interviewee’s perspective, identification of the taxon (like chaff); products made from plants (such as bread, bread mold, vodka, food oil of any origin, etc.) given that the specific species used for their preparation are not mentioned by the interviewee; all animal parts (fat, meat) and products (milk, honey); as well as all other medicaments not purchased from a pharmacy for medicinal purposes nor prescribed by a doctor.

### Region

**Belarus** is located between Lithuania, Latvia, Russia, Ukraine and Poland, in Central and Eastern Europe. It covers a territory of 207.6 thousand sq. km. The climate is moderately continental, transitional between maritime and continental, as it is positioned in the temperate latitudes of the western part of the East European Plain [14]. The Chernobyl disaster, which happened 30 years ago, affected Belarus more than any other country, causing high radioactivity and extensive relocation of people.

Temporal changes in home-gardening in Belarus.

Until the eighteenth century the main vegetable crops grown in the Belarusian lands were onions, cucumbers, cabbage, beets, turnips, carrots, poppy, garlic, parsley, parsnips, and hops, among others. During the second half of the eighteenth century on manor farms people began to plant potatoes, strawberries, beans, cauliflower, swede and spinach. Gardening was poorly developed and apples, plums, gooseberries, and currants were primarily grown. In aristocratic parks and greenhouses, however, rare species of fruit trees and shrubs, such as oranges, peaches, grapes, etc. [15], were planted. The lack of necessary data in the sources of the eighteenth century does not allow determining the place occupied by vegetable growing and gardening in the life of Belarusian peasants [16].

In the nineteenth century vast acres of land were occupied by the cultivation of lentils, beans, and peas [17]. In the Minsk region hops, mustard, coriander, melons and pumpkins, mint, radishes, onion and garlic, lettuce, parsley, poppy, asparagus and tobacco were actively planted [18]. During this period gardens occupied a peripheral place in the economic life of the peasantry [19]. Home-gardens were located close to the house, merging it with housekeeping, and so the garden was almost under sole control of women [20]. The small size of the yard limited the range of cultivated crops to only the most essential ones [19]. At the end of the nineteenth century most of the yard was occupied by hemp and barley, which, according to peasants, were more useful than vegetables [21]. Hemp was cultivated for its fiber, seeds and oil, as well as for medical purposes. At the end of the nineteenth century the cultivation of hemp was one of the basic sources of income of peasants in the provinces of Mahileu and, in part, Minsk [22]. Barley was cultivated as a cereal crop for food, brewing material and animal fodder [23].The most common cultivated plants included cabbage, cucumbers, onion and beets. Occasionally pumpkins, radishes, turnips and beans were also grown [21]. Kidney beans were largely unknown to peasants in the nineteenth century; and mixed crops were a distinctive feature of peasant gardens [21]. Peasant women from suburban villages grew leafy vegetables, such as lettuce, green onion, dill, sorrel, parsley, spinach, etc., which were sold in nearby towns [24, 25]. But above all, people engaged in horticulture to provide for the needs of the family [26]. The cultivation of early vegetables in greenhouses was still not widespread in the nineteenth century [21]. The traditional fertilizer was cattle manure [26].

The twentieth century brought along changes in the structure of the workforce division in villages; however, home-gardening remained one of the primary means of acquiring food for local inhabitants, and even now horticulture remains one of the most important agricultural practices in Belarus (on farms and estates of burghers, village dwellers and even summer residents).

In the researched region literally everyone in the village able to hold a spade keeps a home-garden, which is a relatively small plot close to the house. Most of the space in such gardens is covered by vegetables and fruiting trees, while flower gardens are worth mentioning only when talking about bigger villages. This is quite understandable as the choice of food available in shops is rather limited and is not oriented toward vegetables or fresh food. However, retail shops are present in larger villages and inhabitants of smaller villages are supplied by mobile shops that visit twice a week. Moreover, food is relatively expensive and collective farm workers do not earn much money. For these reasons many villagers still keep domestic animals (pigs, goats, ducks, hens, cows and horses), although admittedly their numbers have diminished during the last several years.

### Data collection

**Fig. 1:**
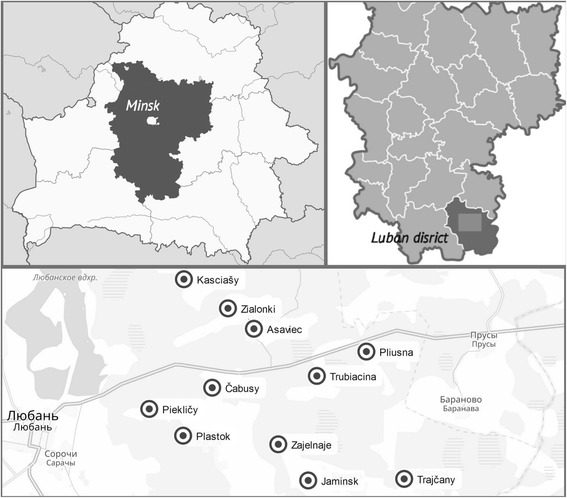
Field research site

The portion of the data contributing to this paper was obtained during a wider ethnomedicinal field study conducted in 11 villages of the **village council of Asaviec**, Liubań district (Fig. 1) in May 2016 as a practical part of a development cooperation project financed by the Ministry of Foreign Affairs of Estonia. The same 134 interviews with locals (born in the villages where the interviews took place or elsewhere in the district of Lubań), as in [13], were selected for this analysis. About two-thirds of the interviewees were women and one-third men, which was due to the low representation of elderly men in the villages; the mean age of the interviewees was 63 years, while the oldest interviewee was 92 years old and the youngest was 27 years old. For more information on the researched area and further details of the study see [13].

This research documented currently used home-remedies as well as those used in the past by employing the folk history method, sensu [27] (reconstruction of historical events through the memory of common people). The subject was approached through specific uses, not by way of remedies. The first part of the interview concerned the use of wild plants and local foods, and this may have introduced little bias as some people paid more attention to wild plants. Yet, in the second part of the interview the domestic sphere of healing was approached through specific illnesses, emphasizing that researchers were interested in all possible means used for healing. In the semistructured format, interviewees were asked to recall what remedies were used to treat most common disease categories using layperson terminology, such as respiratory and cold-related diseases (cold, sore throat, rhinitis, earache, etc.), neurological diseases (headache, nervousness, etc.), cardiovascular ailments (heart problems, blood pressure, etc.) gastrointestinal complaints (stomach ache, diarrhea), musculoskeletal disorders (foot ache, rheumatic diseases, etc.), dermatological conditions (wounds, burns, cuts, skin diseases, etc.), ophthalmological issues (improving vision, eye diseases), oral and dental care (aching tooth, mouth diseases), nephrological ailments (kidney diseases), infection and uses related to other health concerns and wellbeing (prophylactics, being healthy) as well as cosmetic issues (hair and skin care, disinfection, good scent, etc.). After the interviewee responded, a control question was asked, namely if he or she could recall any other specific diseases and their treatment. The veterinary question was addressed more broadly (when animals get ill or how to keep them healthy). Interviewees were allowed to elaborate on the subject as long as they stayed within the health-remedy framework. The interviewees were asked to state when they have used the named remedies and if they have used them personally.

Interviews were voice-recorded upon permission of the interviewee; field notes were also taken and in the majority of cases names recorded, according to the rules prescribed by the local institution. The purpose of the study was explained to each person and prior informed consent was obtained from all interviewees. The Code of Ethics of the International Society of Ethnobiology [28] was followed. All interviews were then transcribed and anonymized before further analysis. The original voice-recorded interviews as well as their transcripts are stored at the The Center for Belarusian Culture, Language and Literature Research within the Archives of The Institute of Art, Ethnography and Folklore, named after K. Krapiva (AIAEF 23–16-2). Anonymized copies of interview transcripts are also stored at the Scientific Archive of the Estonian Folklore Institute (EFISA Valgevene2016) located in the Estonian Literary Museum.

General ethnobotanical practice does not recommend collecting cultivated plant vouchers, therefore all the plants were identified on the spot and only a limited number of voucher specimens were taken to ensure precise identification; in some cases, where people willingly shared their stores, dried plant samples were also archived. Collected voucher specimens were dried and identified with the help of Toomas Kukk (Curator of the Estonian University of Life Sciences herbaria); vouchers are deposited at the Estonian University of Life Sciences herbaria (TAA), assigned herbarium numbers within the range TAA0132555–0132710, and also bearing numbers LJUB001–152. Dried plant samples collected from respondents are deposited at the Scientific Archive of the Estonian Folklore Institute (EFISA Valgevene2016, bearing numbers LJUD001–085). Taxonomic identification, botanical nomenclature, and family assignments of cultivated plants followed the Flora Europaea [29], The Plant List database [30], and the Angiosperm Phylogeny Group IV [31].

Non-plant remedies were identified on the basis of the name used by the interviewees, which as a rule was sufficient for precise identification. Only in few cases were some additional clarification questions asked.

### Data analysis

Anonymized records were entered into a Microsoft Excel spread sheet. As with wild plants [13], to follow *emic* categories, information was structured in Detailed Use Records (DUR adopted from [32]), where interviewees (*i*) mention a specific use (*u*, e.g. emic disease/illness category [cough, sore throat, heart disease, back pain, etc.], emic veterinary treatment) of a remedy or plant part (*p*, e.g. fruits, leaves, aerial parts, flowers, etc.) prepared in a certain way (*w*, e.g. topical application of the remedy, tea [plants macerated in hot water], decoction [plants boiled in water], tincture [plants or other substances macerated in alcohol – either applied or drunk], special preparation, etc.). Interviewee-defined emic categories were employed to ascertain local perceptions.

For every taxon, the number of Use Citations (UC – number of people who claimed the (specific) use of the remedy during the interview) and the number of DUR were calculated for the sum of all uses and separately for medicinal and veterinary areas. For general and emic disease/illness categories, Use Instance (UI), representing the attribution of one specific taxon to a disease category, regardless of the number of people mentioning the specific use, was calculated. Following the recommendation given in several recent publications [33, 34] and to illustrate the diversity of various uses, uses mentioned by only one person were also included.

An Informant Consensus Factor (FIC [35]) was calculated for both remedy groups (cultivated plants and non-plant remedies) as well as for different use categories within each of the groups.

- FIC = nUC – nT / (Nuc – 1).
- nUC = number of use citations for a particular plant-use category;
- nT = number of taxa or species that are used for that plant use category.
- FIC values range between 0 and 1, where ‘1’ indicates the highest level of informant consent.

The reliability criterion [36] was also assessed to detect the proportion of remedies used by more than three people.

On a temporal scale all uses were divided into four blocks: 1) continuously used (reported to be used throughout life), 2) past uses (uses that were talked about in the past tense), 3) temporary uses (practiced for the limited period of time during adulthood), and 4) adulthood uses (practices that were not known to the person during childhood, but picked up at some point in adulthood and still practiced). The issue of the temporal scale bears some limitations as not all people responded to the question regarding the time in which a remedy was used in an unambiguously interpretable way (and at times the interviewers were too engrossed in the conversation to ask clarifying questions). For about one-third of the use reports, attribution to the temporal block was made on the basis of context (verb tense the person used, time period they were talking about, etc.) and, therefore, this scale reflects in part the approach of the researcher. However, as all researchers collecting data asked the same questions about the same diseases treated, even a slightly vague interpretation of the temporal scale deserves to be analyzed.

#### Qualitative comparison with historical uses and quantitative comparison with medicinal and ethnoveterinary uses of wild plants

The peak of research on the ethnomedicine of Belarus occurred at the end of the nineteenth century [36].

In those publications special attention was paid to the causes of illnesses, their prevention, and ways of ritual treatment, but the comments were developed in accordance with the attitudes of the collectors - i.e. to show the “downtrodden” peasants or romanticize their “antiquity”. Representatives of the mythological school enthusiastically talked about the “ghosts and demons” of diseases and ways of communicating with them [18, 26, 37, 38]. By the middle of the nineteenth century, among the collectors and researchers of folk customs, there was already the idea of a separate sphere of national knowledge - “folk medicine”. Materials on folk medicine were systematically collected and discussed in publications [37–46]. Folk medicine can be understood from two different perspectives: 1) to reveal the reasons for the stability of folk techniques and 2) to enrich medical science with unknown medicines. The common tendency in evaluating traditional medicinal practices was to consider “superstitious” techniques (from the collectors’ point of view) as “age-old backwardness” and “useful” ones as “folk wisdom and experience”.

Special attention was also paid to the study of medicinal plants. At the end of nineteenth century ethnographers encouraged the creation of herbariums of local flora. For example, E. Orzeszkowa established a full herbarium of the vegetation of the Neman river region, in which the specimens were accompanied by a description of their uses in folk medicine, as well as related proverbs and beliefs [47]. The historical sources also allow us to see a more diverse picture of the cultivated plants and non-plant remedies employed in ethnoveterinary medicine in the 19th and early 20th centuries [48–56].

The historical vicissitudes of the twentieth century and changes in the scholarly approach to ethnomedicine [57, 58] did not allow for the gathering of much comparative data from that period.

In Soviet times there were almost no folkloric or ethnographic studies on the subject of folk medicine, which seems to indicate the absence of official support for such documentation at that time. At the end of the twentieth century publicatons focused on the ethno-cultural component of folk healing and the mythology of diseases. The first volume of the “Polack Ethnographic Collection” (Issue 1, "Folk Medicine of Belarusians from Padzvinnie region") [59] was dedicated to folk medicine. Summarizing the results of a study was the volume “Traditional medicine and ritual-magic practices” prepared by T.Valodzina from the series “Belarusian Folk Art” [37], which presents a chronologically blurred section of Belarusian magical healing. This volume is a complete compendium of folk medicinal ritual-magical practices, collected from a variety of printed sources and archives. A large part of the material was collected by the author during more than 100 expeditions between 1993 and 2010 to all regions of Belarus under a special program (more than 7000 folklore units were recorded).

As of today, Polish scientists have reviewed the unpublished sources of the late nineteenth century on ethnobotany, including the Belarusian lands: a questionnaire by Yu. Rostafinski (1883) [60] and a manuscript by M. Federowski, who structured his data in accordance with the earlier questionnaire of Rostafinski [61].

Therefore, qualitative comparisons with the current collected data and historical records covering all of Belarus can be drawn for both human and animal medication, especially when it concerns non-plant remedies.

The results obtained for cultivated plants and remedies were compared with similar results for wild plants as in [13], and a few additional aspects (like the quantification of recently acquired uses) of the data regarding the use of wild food plants were further analyzed and added to the already published results. In this instance, wild plants consisted of all plants growing outside direct human cultivation, including domesticated local wild plants cultivated in home gardens, but not for food purposes (such as bedding plants and non-fruiting trees, which can also be encountered outside of human cultivation).

## Results and discussion

### Human medication

#### Cultivated plants

Interviewees used 45 cultivated plant taxa belonging to 27 families (Table 1). The most well represented families were Lamiaceae (six taxa), Rosaceae (five taxa), Poaceae (four taxa), Solanaceae and Asteraceae (three taxa each). The reliability criterion was met by 26 taxa (57%).

**Table 1:** Medicinal and ethnoveterinary uses of cultivated plants

Plant taxon, family (voucher specimen code)	Local names	Used part(s)	Preparation	Recorded medical use(s) (treated disease)	UC
*Beta vulgaris* L., Amaranthaceae	буракі, свякла / buraki, sviakla	roots	fresh	laxative	3
				constipation	2
			fresh, added to fodder	fodder for cows, increase milk production	1
		tubers	juice drunk	constipation	1
*Allium cepa* L., Amaryllidaceae	лук, цыбуля / luk, cybulia	bulbs	baked	rhinitis	1
			baked, topical application	rotten wounds	1
				abscesses	3
				wounds	1
			boiled with milk	cough	1
			fresh	helmitic infection	6
				rhinitis	1
			inhalation	rhinitis	2
				wellbeing	1
			topical applicaton	skin diseases	2
				snake bites	1
		whole plant	fresh	immune boosting	1
			tea	panacea	1
*Allium sativum* L., Amaryllidaceae (LJUD037)	часнык, чэснок / časnyk, česnok	bulbs	fresh	helmitic infection	7
				cold	2
				immune boosting	2
				cough	1
				rhinitis	1
			alcohol maceration, topical application	joint pain	1
			necklace	cold	1
				prophylactics	1
			steamed with milk	helmitic infection	1
			tea	panacea	1
			topical applicaton	toothache	5
				helmitic infection	1
				rhinitis	1
		fruits	fresh	cancer	1
		seeds	decoction	good for pigs (vet)	2
				rumination problems in cows (vet)	2
*Anethum graveolens* L., Apiaceae (LJUD014)	кроп, укроп / krop, ukrop	aerial parts	boiled	diarrhea in cows and pigs (vet)	1
			decoction	rumination problems in cows (vet)	1
			dried	spices	1
			fresh	spices for cucumbers	7
				soup	5
				spices	2
				spices for soup	2
				salad	1
				spices for sauerkraut	1
		seeds	decoction	increase cow milk production	1
			dried	spices	11
				spices for meat	4
				spices for sausages	3
				spices for soup	3
				condiment	1
				spices for pork fat	1
				spices for potatos	1
				spices for salad	1
				flatulence	1
				stomachache	1
			tea	stomachache	4
				burns	1
				flatulence in children	1
*Petroselinum crispum* (Mill.) Fuss, Apiaceae (LJUB134)	пятрушка / piatruška	aerial parts	fresh	men’s health	1
		leaves	fresh	potency	1
*Albuca bracteata* (Thunb.) J.C.Manning & Goldblatt, Asparagaceae	лук індзійскі, кітайская цыбуля, вазон / luk indzijski, kitajskaja cybulia, vazon	leaves	alcohol maceration	wellbeing	1
				applied on aching joints	1
				applied against reumatic pains	1
			topical applicaton	burns	2
*Calendula officinalis* L., Asteraceae (LJUD032)	нагoткі, календула / nahotki, kaliendula	aerial parts	compress	eye problems	1
			tea	sore throat	1
				stomachache	1
			topical applicaton	cuts	1
		flowers	decoction	gingival inflamation	1
				hair care	1
			alcohol maceration	heart problems	1
			tea	stomachache	1
*Helianthus annuus* L., Asteraceae	падсолнечнiк / padsolniečnik	seeds	fresh oil	constipation	1
			oil given	rumination problems in cows (vet)	2
*Helianthus tuberosu*s L., Asteraceae	груша земляная, тапiнамбур, ціпанамбур / hruša ziemlianaja, tapinambur, cipanambur	flowers	tea	diabetes	1
		tubers	fresh	diabetes	1
			fresh, fodder	good for home animals (vet)	1
*Brassica oleracea* L., Brassicaceae	капуста, капуснік / kapusta, kapusnik	brine	feeded	rumination problems in cows (vet)	2
			drunk	constipation	2
		leaves	topical applicaton	joint pain	6
				headache	5
				inflamation after injection	1
				reumatic pains	1
*Raphanus raphanistrum* subsp. sativus (L.) Domin, Brassicaceae	редзька чёрная, рэдзіска / riedźka čiornaja, redziska	roots	eaten fresh	wellbeing	1
				kidney stones	1
				rhinitis	1
*Cannabis sativa* L., Cannabaceae	канапля / kanaplia	seeds	eaten fresh	constipation	1
*Callisia fragrans* (Lindl.) Woodson, Commelinaceae (LJUB153)	зелёный ус, залатой ус / zielionyj us, zalatoj us	aerial parts	alcohol maceration, topical application	joint pain	1
			tea	cancer	1
*Kalanchoe* spp., Crassulaceae	каланхое / kalanchoje	leaves	tea	sore throat	1
			topical applicaton	wounds	1
		sap	drops into nose	rhinitis	1
*Cucumis sativus* L., Cucurbitaceae	агуркі / ahurki	fruits	fermented	rumination problems in cows (vet)	2
				appetizer for animals (vet)	1
*Cucurbita pepo* L., Cucurbitaceae	гарбуз, тыква / harbuz, tykva	fruits	fresh	fodder for pigs (vet)	1
		seeds	dried	helmitic infection	2
			fresh	helmitic infection	2
*Hippophae rhamnoides* L., Elaeagnaceae (LJUB126)	абляпіха / abliapicha	fruits	jam	cold	1
			oil	burns	3
*Pelargonium* spp., Geraniaceae (LJUB112)	герань / hierań	leaves	fresh into ear	earache	2
			inbetween cloth	moth repellent	2
			topical applicaton	sedative	2
				headache	2
*Ribes nigrum* L, Grossulariaceae	смародзіна, чорная смародзіна / smarodzina, čornaja smarodzina	fruits	fresh	diarrhea	1
				hypertension	1
		leaves	tea	cold	1
				diuretic	1
				wellbeing	1
				immune boosting	1
				sore throat	1
		shoots	tea	cold	1
				strenghtening of organism	1
		twigs	bath	diathesis in children	1
			decoction	cold	1
*Ribes uva-crispa* L., Grossulariaceae	aгрэст, крыжоўнік / ahrest, kryžoŭnik	fruits	alcohol maceration	soporific	1
				stomachache	1
*Hydrangea* spp., Hydrangeaceae	гартэнзія / hartenzija	leaves	topical applicaton	earache	1
*Hyssopus officinalis* L., Lamiaceae	iсоп / isop	leaves	tea	cough	1
				stomach problems	1
*Leonurus quinquelobatus* Gilib., Lamiaceae (LJUB004)	пустырнік / pustyrnik	aerial parts	alcohol maceration	heart problems	1
			decoction	heart problems	2
			tea	sedative	1
				diarrhea	1
		leaves	tea	heart problems	2
				helmitic infection	1
		root	alcohol maceration	heart problems	1
*Melissa officinalis* L., Lamiaceae	меліса лімонная, мята лімонная, лімонка, мята перачная / mielisa limonnaja, miata limonnaja, limonka, miata pieračnaja	leaves	tea	wellbeing	1
				stomachache	1
*Mentha* spp., incl. *Mentha ×piperita* L., Lamiaceae (LJUB011)	м’ята / mjata	aerial parts	decoction	sedative	5
			tea	cough	2
				headache	1
				heart problems	2
				hemorroids	1
				sedative	1
			topical applicaton	wounds	1
*Nepeta cataria* L., Lamiaceae (LJUB072)	меліса / mielisa	aerial parts	tea	sedative	1
				cancer	1
				kidney diseases	1
				panacea	1
				stomachache	1
*Salvia officinalis* L., Lamiaceae (LJUB025)	шалфей / šalfiej	aerial parts	tea	panacea	1
		leaves	tea	toothache	1
				diarrhea	1
*Vicia faba* L., Leguminosae	боб, фасоль / bob, fasoĺ	beans	boiled	diarrhea	1
				diarrhea in cows (vet)	1
*Linum usitatissimum* L., Linaceae	лён / lion	seeds	boiled	diarrhea in cows (vet)	7
				diarrhea in pigs (vet)	5
			decoction	rumination problems in cows (vet)	8
				good for pigs (vet)	2
			dried	constipation in cows (vet)	1
			oil	men’s diseases	1
			tea	stomachache	2
*Althaea officinalis* L., Malvaceae	алтэй / altej	aerial parts	tea	cold	1
				sedative	1
			fed fresh	rumination problems in cows (vet)	2
*Paeonia* spp., Paeoniaceae	піон укланяюшчійся / pion uklaniajuščijsia	roots	alcohol maceration	sedative	1
*Cedrus*spp., Pinaceae	кедр / kiedr	nuts	dried	macerated in strong alcohol	1
*Piper nigrum* L., Piperaceae	перац горкі гарошкам, перац чорны / pierac horki haroškam, pierac čorny	seeds	fed	weakness in chickens (vet)	1
			fresh	diarrhea	2
			mixed with alcohol, drunk	cold	2
				rhinitis	1
*Avena sativa* L., Poaceae	авёс / avios	shoots	decoction	liver diseases	2
		grains	decoction	cold	1
*Oryza sativa* L., Poaceae	ріс / ris	grains	decoction	diarrhea	3
*Zea mays* L., Poaceae (LJUD018)	кукуруза / kukuruza	silk	tea	liver diseases	1
*Aronia* spp. (*Aronia* × *prunifolia* (Marshall) Rehder (LJUB033) and *Aronia melanocarpa* (Michx.) Elliott), Rosaceae	aронія, рабіна чорная, рабіна чарнаплодная, чарнаплодка / aronija, rabina čornaja, rabina čarnaplodnaja, čarnaplodka	fruits	boiled	diarrhea in pigs (vet)	1
			dried	diarrhea	2
				panacea	1
			fresh	hypertension	2
				hyperthyroidism	2
				diarrhea	1
			alcohol maceration	hypertension	2
				wellbeing	1
			tea	diarrhea	2
				hypertension	1
				liver diseases	1
		leaves	tea	hypertension	1
*Chaenomeles japonica* (Thunb.) Lindl. ex Spach, Rosaceae	aйва японская / ajva japonskaja	fruits	jam, raw	wellbeing	1
*Prunus cerasus* L., Rosaceae (LJUB097)	вішня, вішняк / višnia, višniak	leaves	tea	sore throat	1
		twigs	tea	cold	2
				strenghtening of organism	1
*Pyrus communis* L., Rosaceae	груша / hruša	fruits	dried	diarrhea	4
*Citrus limon* (L.) Osbeck; Rutaceae	лімон / limon	fruits	fresh	cold	1
*Capsicum annuum* L., Solanaceae (LJUD015)	красный перец / krasnyj pieriec	fruits	alcohol maceration, topical application	joint pain	1
*Nicotiana* spp., Solanaceae	табак / tabak	leaves	tea, grugling	toothache	1
*Solanum tuberosum* L., Solonaceae	бульбa, картофель, картошка / buĺba, kartofieĺ, kartoška	flowers	topical applicaton	joint pain	1
		tubers	boiled	diarrhea in calves (vet)	1
			candles	hemorroids	3
			compress	eye inflamation	2
			fresh, fodder	for cows, produce more milk	2
				fodder for pigs	1
			smashed hot, applied on chest	cough	1
			inhalation of boiling water	rhinitis	5
				cold	2
				cough	1
			topical applicaton of fresh slices	burns	3
				skin diseases	2
*Camellia sinensis* (L.) Kuntze, Theaceae	чай (крепкі), чай чорны пакупны / čaj (kriepki), čaj čorny pakupny	leaves from shop	compress	eye problems	7
			decoction	eye inflamation in cats (vet)	1
			eaten dried	diarrhea	1
			tea	diarrhea	1
*Vitis* spp., Vitaceae	вінаград / vinahrad	fruits	fresh	hypotension	1
*Aloe* spp. (mainly *Aloe arborescens* Mill.), Xanthorrhoeaceae	альвас, гальяс, aлоэ, сталетнік / aĺvas, haĺjas, aloe, stalietnik	leaves	eaten fresh, mixed with honey	cold	3
				stomachache	1
			alcohol maceration	lung diseases	3
				tuberculosis	1
			topical applicaton	wounds	3
				burns	2
		sap	cooked	lung diseases	1
			drunk fresh	cold	1
			topical applicaton	inflamation	1
				cuts	1
				joint pain	1
				rotten wounds	1
				wounds	4
			washed with	beauty procedure	1

Two hundred twenty-six DUR of cultivated plants were recorded. The six most widely utilized taxa (and named by at least 10% of interviewees) included two *Allium* taxa (garlic *A. sativum* [26 DUR] and onion *A. cepa* [22 DUR]), followed by houseplant aloe *Aloe* spp. (24 DUR), potato *Solanum tuberosum* (20 DUR), aronia *Aronia spp.* (16 DUR), and cabbage *Brassica oleracea* (15 DUR).

Altogether 108 UI were detected in general disease categories. The reliability criterion (at least three users) was met by 30 general UI. This group contained 19 taxa, of which only seven were used in more than one wider disease category, including garlic *Allium sativum* and onion *Allium cepa* in four disease categories, three of which (respiratory, general health and infection) were shared, while the other two were oral and dental and dermatological, respectively. Potato *Solanum tuberosum* was used in three general categories (dermatological, respiratory and gastrointestinal). The remaining four taxa met the reliability criterion in two disease categories: blackcurrant *Ribes nigrum* and black pepper *Piper nigrum* (respiratory and gastrointestinal in both), aloe *Aloe* spp. (respiratory and dermatological) and aronia *Aronia* spp. (gastrointestinal and cardiovascular). Among the other twelve taxa, the most well represented included ophthalmalogical use of leaves of the tea plant *Camellia sinensis*, gastrointestinal use of dill *Anethum graveolens* and *Beta vulgaris*, neurological use of mint *Mentha* spp. and cardiovascular use of *Leonurus quinquelobatus*, all reported by at least five people.

Individual use of cultivated plants was not very diverse. Only one person claimed to have used one taxon, namely calendula (*Calendula officinalis*), to treat five different illness categories, while three more taxa (*Anethum graveolens*, *Solanum tuberosum* and *Mentha* spp.) were used by a single individual in three different illness categories.

The Informant Consensus Factor for the entire medicinal use area was relatively low at 0.82 (251 use citations for 45 taxa). All of the general categories in the medicinal use area had lower FIC values and only five general categories had a FIC greater than 0.6.

**Fig. 2:**
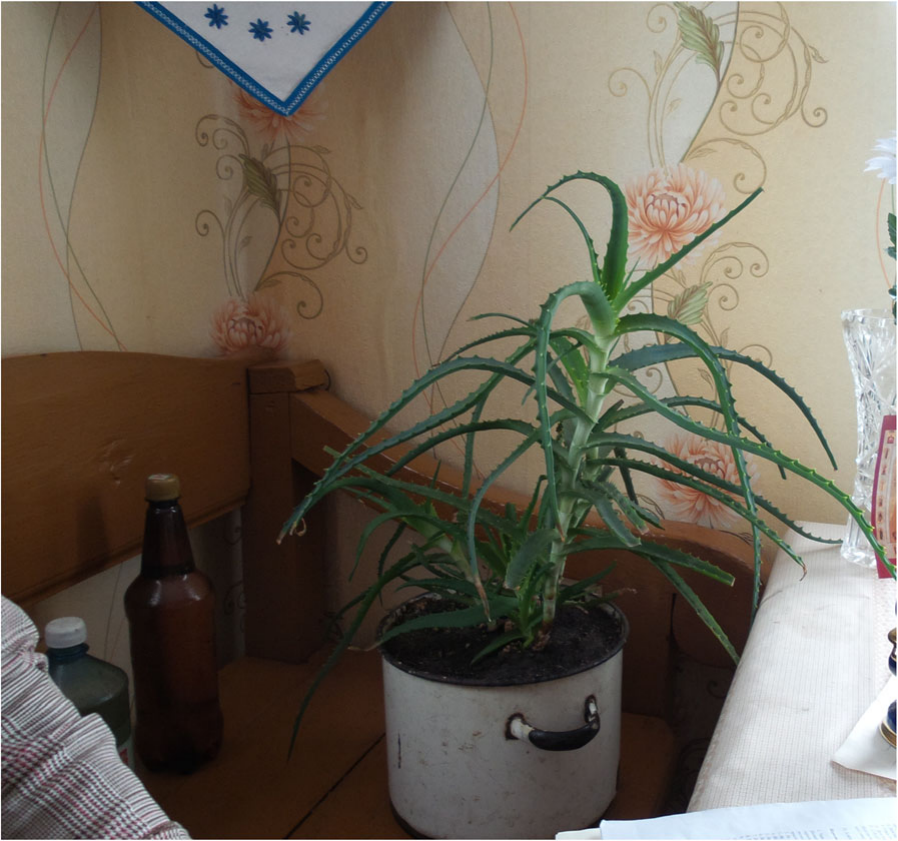
Home-grown aloe (*Aloe arborescens*). Plastok village, photo Renata Sõukand. 13.05.2016

The disease category with the highest FIC was **infection** (FIC 0.79 for five taxa), which was dominated by two *Allium* taxa and *Cucurbita pepo* used to treat helminthic infection. Another small category with only three taxa used was **ophthalmological** (FIC = 0.77), which was dominated by *Camellia sinensis* (used only in this disease category to treat eye problems). The **dermatological category** (FIC 0.69, ten taxa) was clearly dominated by the taxon *Aloe* spp. (Figure 2), which was used to treat mainly wounds, but also burns, cuts and skin inflammation. Only a few other taxa in this diease category met the reliability criterion: *Allium cepa* (used to treat abscess, (rotten) wounds and skin diseases), *Hippophae rhamnoides* (oils for treating burns), and *Solanum tuberosum* (for treating burns and skin diseases).

Only two taxa in the **cardiovascular** category (FIC = 0.64, six taxa) met the reliability criterion: *Aronia* spp. to treat hypertension and *Leonurus quinquelobatus* to treat heart problems. The second most numerous in terms of represented taxa was the **respiratory** category (FIC = 0.63, 18 taxa), which consisted of six taxa, none of which were clearly dominate, that met the reliability criterion: *Aloe* spp. (eaten fresh against cold and macerated in alcohol or cooked to treat lung diseases or tuberculosis (in the past)), *Solanum tuberosum* (used as an inhalation against cold, cough and rhinitis), *Allium sativum* (eaten fresh, worn as a necklace to combat cold and cough, and applied topically to alleviate rhinitis), *Allium cepa* (boiled with milk or inhaled to treat cough and rhinitis), *Ribes nigrum* (used as a tea against cold) and *Prunus cerasus* (used as a tea to combat cold and sore throat).

The **gastrointestinal** category, while having a relatively low FIC value (0.59 for 25 taxa), contained seven taxa that met the reliability criterion: *Anethum graveolens* (to alleviate flatulence and stomach ache)*, Aronia* spp. (to relieve stomach ache)*, Beta vulgaris* (to cure constipation), *Pyrus communis* and *Oryza sativa* (to treat diarrhea), *Piper nigrum* (to treat diarrhea and stomach ache), *Solanum tuberosum* (to treat hemorrhoids). Within the other disease categories only a few individual taxa met the reliability criterion, among these: *Allium sativum* in the **oral and dental** category (to treat toothache); *Mentha* spp. (as a sedative and to relieve headache) and *Brassica oleracea* (against headache) in the **neurological category**; *Brassica oleracea* (to alleviate joint pain) in the **musculoskeletal** category; and *Allium sativum* and *Ribes nigrum* in the **general health** category.

The majority of the temporary, past and adulthood uses of cultivated plants overlap with uses that have been continuously practiced; however, some differences do exist. For example, some plants were reported to be used differently in the past than they are now, including the tea of cultivated *Mentha* species which in the past was drunk to treat hemorrhoids, but now is used in adulthood by many people as a sedative (also used continuously) and to treat respiratory illnesses. The use of *Mentha* leaves applied on wounds was also only practiced in the past. However, uses of cultivated plant taxa practiced only in the past were rather rare and included the freshly pressed oil of *Cannabis sativa* which was drunk to cure constipation and the tea of *Nicotiana* spp. which was gurgled to alleviate toothache. A few additional taxa were mentioned only when talking about uses acquired during adulthood; for example, *Capsicum annuum* macerated in alcohol to treat rheumatic diseases or raw jam of *Chaenomeles japonica* to promote wellbeing, roots of *Helianthus tuberosus* eaten raw to treat diabetes, and tea of the leaves of *Hyssopus officinalis* to combat cold and stomach problems – all of which are known from the widespread popular literature on home-medication and widely promoted in the media (for example, the most popular newspaper “*Narodnyy Doktor*”publishes readers’ recipes that are sent to the editorial office).

One temporary use also stood out, although the plant itself was continuously used: children were bathed in a decoction of twigs and leaves of *Ribes nigrum* against diathesis. The person describing the use mentioned that the wild plant (three-lobe beggarticks, *Bidens tripartita* L.) did not help her children, “but the leaves of blackcurrant did”.

Out of the 134 people interviewed, 73 mentioned using cultivated or store-bought plants for medication. Twenty-five people mentioned only one use, while the most knowledgeable person mentioned 16 taxa and three more individuals recalled 13 taxa. The mean number of used taxa was 3.6, which is relatively quite low, especially considering that almost all households had a vegetable garden, indicating that plants were cultivated.

##### Comparison with the available historical data

While the historical data on the use of cultivated plants for healing is relatively limited, some comparisons can be drawn. For example, the use of intensely tasting plants have retained their popularity, even if the specific use indicated has changed considerably in nature: at the end of nineteenth century it was believed that onion and garlic would frighten off diseases, as well as protect children and pregnant women, and also newlyweds during their wedding [40, 43, 44, 62], whereas now only a few interviewees recalled the use of garlic (as a necklace) as a means of maintaining good health and protecting against cold, while the primary emphasis of the modern use of fresh onion and garlic is on respiratory diseases. In the nineteenth century garlic was used to relieve teething pain in infants [43, 62], while now it is used to treat toothache. The specific use of baked onions has also changed as now it is used mainly to treat dermatological problems, while according to nineteenth century sources mothers used to rub the new-born’s breast with baked onions to alleviate rhinitis [63].

### Non-plant remedies

There were 188 DUR referring to the use of 58 different remedies (Table 2). The six most popular remedies included vodka (19 DUR), pork fat and honey (both 14 DUR), salt (11 DUR), propolis (10 DUR), and goat milk (9 DUR); however, only vodka was named by more than 10% of interviewees.

**Table 2:** Non-plant remedies used to treat humans and other animals

Remedy	Local names	Preparation	Application	UC
Anthill	*куча мурав’ёф / kuča muravjof*	ritual, literature based, bath	epilepsy	1
Beer	*піва / piva*	forced to drink	rumination problems in cow (vet)	3
		poured on stones in sauna	aromatherapy	1
Bile of the pig	*жэлчь ат свіней / želč́ at sviniej*	macerated in alcohol, topical application	pain	1
Brandy	*каньяк / kańjak*	drunk	kidney stones	1
Bread	*хлеб / chlieb*	mumbled on, allowed to eat	to protect cows against snake bites (vet)	1
			to protect cows (vet)	1
Bread, soft part of it	*мякіш хлеба / miakiš chlieba*	roll over the stomach, say words and then fed to a dog	fright	1
Bread mold	*цвілы хлеб / ćvily chlieb*	scratched from bread, mixed with fat, smeared	mastitis in cows (vet)	1
Brick	*кірпічына / kirpičyna*	sit on a bucket with hot water and hot brick	women’s diseases	1
Broken hay, chaff	*дзёран / dzioran*	fodder	vitamins for calves (vet)	2
Butter	*масла / masla*	heated until boiling, eaten	diarrhea	1
Chicken navel skin	*плёначкі ад курыных пупкоў / plionački ad kurynych pupkoŭ*	dried, macerated in hot water	diarrhea	1
Clay	*гліна / hlina*	fresh, fodder	given to calves (vet)	2
		diluted with water, forced to drink	when an overheated horse has drunk cold water (vet)	1
		fresh, additional fodder	pigs, to make joints strong (vet)	1
Cow milk, freshly milked	*сырадой / syradoj*	washed with	eye inflammation	1
Diesel oil	*дзізельнае масла, аўтол / dzizieĺnaje masla, aŭtol*	forced to drink	rumination problems in cow (vet)	1
		topical application	skin diseases in pigs and piglets (vet)	1
Dog hair	*шэрсць сабакі / šersć sabaki*	cut and set on fire	fright	2
Dung water or horse feces	*конскі гной, конскі кал / konski hnoj, konski kal*	dropped on sugar, eaten	helminthic infection	1
		fed	helminthic infection in pigs (vet)	3
		few drops put into glass of water, so person drinking it does not know	helminthic infection	3
Egg	*яйка / jajka*	boiled, applied hot	allergic rhinitis	1
		boiled, applied hot	sinusitis	1
		boiled	diarrhea (in children)	2
Egg and vodka	*яйцо з гарэлкаю / jajco z harelkaju*	mixed and forced to drink	diarrhea in piglets (vet)	3
Egg yolk	*яічный жэлток / jaičnyj želtok*	salve made with oil of *Helianthus annuus* and egg yolk	panacea	1
Egg white	*бялок яйца / bialok jajca*	applied fresh	burns	1
Fat of the badger	*барсучья сала / barsuč́ja sala*	melted and smeared	burns	1
Fat of the goose	*гусіны жыр / husiny žyr*	mixed with honey and applied with cabbage leaves on chest	cough in children	3
Fat of the pig	*сала / sala*	fresh, topical application	foot sores	1
		fresh, topical application	toothache	5
		salted, topical application	toothache	6
		smeared on scalp	to increase hair growth	1
		topical application	mastitis in cows (vet)	2
		ointment made with turpentine, topical application	aching legs	1
Fat of the ram	*барані жыр / barani žyr*	mixed with hot milk or honey and vodka	cough	1
Fat of the sheep	*авечы жыр / aviečy žyr*	added to hot milk	cold in children	1
		salve made with root of Arctium spp.	joint pain	1
Fat of the goose	*гусіны жыр / husiny žyr*	smeared	burns	2
			cold	1
			earache	1
			foot sores	1
Fat of the hedgehog	*вожыка жыр / vožyka žyr*	boiled, smeared	burns	1
Fireplace grime	*сажа з коміна / saža z komina*	forced to eat	diarrhea in calves (vet)	1
Flour	*мука / muka*	fodder additive	to increase milk production in cows (vet)	3
Grasshopper	*конік, кузнечык, цвыркун / konik, kuźniečyk, cvyrkun*	topical application	warts	2
Honey	*мёд / miod*	diluted with water, topical application	eye problems	1
		mixed with goose fat and applied with cabbage leaves on chest	cough in children	3
		eaten	cold	2
			cough	1
			panacea	1
			sore throat	1
			stomach ache	1
		topical application	burns	1
		mixed with hot milk, drunk	cough	1
		mixed with vodka, applied on throat	sore throat	1
Honeybees	*пчала / pčala*	allowed to sting	rheumatism	1
Honeybees, dead	*пчаліны падмур / pčaliny padmur*	macerated in alcohol (for 2 weeks)	heart problems	2
			hypertension	2
			joint pain	1
Kefir	*кефір / kiefir*	drunk	constipation	3
Lactic acid	*малочная кіслата / maločnaja kislata*	forced to drink	rumination problems in cows (vet)	1
Laundry soap	*хазяйсцвеннае мыла / chaziajscviennaje myla*	ground, compress, external	sore throat	2
		ground, clyster	constipation in cows (vet)	3
		smeared	mastitis in cows (vet)	1
		topical application	wounds	1
		smeared	mastitis in cows (vet)	5
Leftovers from moonshine distillation	*брага / braha*	forced to drink	stomach ache in cows (vet)	1
Liquid left after making curds	*сыроватка (сываратка) / syrovatka (syvaratka)*	forced to drink	rumination problems in cows (vet)	1
Lice	*вош / voš*	hidden in bread and given to eat	jaundice	1
Meat of the badger	*барсук / barsuk*	boiled and eaten	tuberculosis	1
Meat of the dog	*сабака* / *sabaka*	dried, eaten	tuberculosis	1
		made into cutlets	tuberculosis	1
		boiled and eaten	tuberculosis	1
Milk of the cow	*малако* / *malako*	garlic added and sit in hot bath	helminthic infection	1
		forced to drink	stomach problems in cows (vet)	1
		heated, soda added, drunk	sore throat	1
Milk of the cow, sour	*кіслае малако* / *kislaje malako*	drunk	constipation	2
Milk of the goat	*казінае малако* / *kazinaje malako*	drunk	allergies in children	3
		drunk hot	cough in children	3
		topical application	eye pain	1
		drunk	cancer	1
		drunk	asthma	1
		heated, drunk with honey	earache	1
			cough	3
		heated, drunk with soda	cough	3
Motor oil	*маторнае масла* / *matornaje masla*	forced to drink	stomach problems in cows (vet)	1
Mussel shells or chalk	*ракушкі ці мел / rakuški ci miel*	broken, fodder additive	to make egg-shells stronger (vet)	1
Oil	*алей* / *aliej*	drunk	constipation	5
		forced to drink	rumination problems in cows (vet)	2
Petroleum	*керасін, кірасін* / *kierasin, kirasin*	compress	sore throat	2
Petrol	*бензін, салярка* / *bienzin, saliarka*	applied on scratched warts	warts	1
		topical application	eczema	1
Potassium permanganate	*марганцоўка* / *marhancoŭka*	drunk	vomiting	1
		weak solution, drunk	diarrhea	1
Propolis	*праполіс* / *prapolis*	macerated in alcohol, topical application	joint pain	4
		macerated in alcohol, drunk	panacea	2
			stomach ache	1
		macerated in strong alcohol	sore throat	1
Rain water	*дажджавая вада* / *daždžavaja vada*	washed with	hair care	1
Salt	*соль* / *soĺ*	dissolved in water, gurgling	toothache	1
		heated, topical application	cystitis	3
			earache	1
			toothache	1
			sore throat	1
		heated and put into socks	cough	1
		mixed with vodka, drunk	diarrhea	1
		mixed with rye flour, heated, topical application	sinusitis	1
		ritual “salting”	evil eye	1
Salted water	*салёная вада / salionaja vada*	side of the calve pierced and the mixture poured into wound	rumination problems in calves (vet)	1
Snakeskin	*змяіная кожа / zmiainaja koža*	dried, topical application	burns	1
Snakeskin (old)	*змеіная лінь (старая шкура) / zmieinaja liń (staraja škura)*	topical application	pimples	1
Sand where chickens bathe	*пясок, дзе куры купаюцца / piasok, dzie kury kupajucca*	topical application	warts	1
Sebaceous secretion of goose	*гусіны гной / husiny hnoj*	smeared on with leaves, topical application	splinter in the finger	1
Snailshells or chalk	*ракушкі ці мел / rakuški ci miel*	broken, fodder additive	to make chicken egg-shells stronger (vet)	1
Soda	*сода / soda*	bath	foot sores	1
		gurgling with solution	sore throat	2
		mixed with water, drunk	cleaning of stomach	1
		mixed with water, eyes washed	eye pain	1
		mixed with water, forced to drink	rumination problems in goats (vet)	1
		topical application	burns	1
Spirits	*спірт / spirt*	drink one teaspoon every day	against a fright from lightening, not to be nervous and nasty throughout life	1
		smeared	sore throat	1
		topical application	cold	1
			eczema	1
			joint pain	1
			psoriasis	1
			sore throat	1
Starch	*крахмал / krachmal*	eaten dry and water drunk afterwards	diarrhea	1
		mixed with cold water, drunk	diarrhea	2
		mixed with cold water, forced to drink	diarrhea in calves (vet)	1
Sugar	*сахар / sachar*	topical application	eye pain	1
			wounds	1
Sweat of horses	*конскі пот / konski pot*	topical application	warts	1
Tar	*дзёгаць, тавот / dziohać, tavot*	smeared	red fever in pigs (vet)	1
			scabs in pigs (vet)	1
Trichlorfon	*хларафос / chlarafos*	diluted, topical application	skin diseases in pigs	1
Turpentine	*шпікідар / špikidar*	topical application on navel; dripped on sugar, eaten	helminthic infection	1
Urine	*мача / mača*	compress	joint pain	1
		drunk	fright	1
		smeared	mastitis in cows (vet)	4
		topical application	cancer	1
			cuts	1
			wounds	1
			wounds in horses (vet)	1
Water, cold	*халодная вада / chalodnaja vada*	topical application	headache	1
Wet cloth	*мокрая трапка / mokraja trapka*	put on cow stomach	rumination problems in cows (vet)	1
Window “sweat”	*пот на вакне / pot na vaknie*	topical application	eczema	1
			burns	1
Vinegar	*уксус / uksus*	topical application	warts	3
Vodka / moonshine	*гарэлка, водка, самагонка / harelka, vodka, samahonka*	applied on hole	toothache	1
		compress	back pain	1
			foot ache	1
			sore throat	1
		drunk	diarrhea	1
			panacea	1
			stomach ache	1
		forced to drink	diarrhea in pigs (vet)	1
			dog plague (vet)	2
			goat kids’ illness (vet)	1
			helminthic infection in dogs (vet)	1
			rumination problems in cows (vet)	4
			stomach ache in cows (vet)	2
		smeared	fever	1
			mastitis in cows (vet)	2
			stomach ache	1
		smeared on soles	cough	1
		gurgled	toothache	1
		salted a bit and drunk	diarrhea	1
		topical application	earache	2
Vodka and pepper	*гарелка з перцам / harielka z piercam*	mixed, drunk	cold	2
			rhinitis	1
			diarrhea	1
			stomach ache	1
Vodka, diluted with human urine	*водка разбавленая челавеческай мачёй / vodka razbavlienaja čielaviečieskaj mačioj*	given to drink	when a person drinks too much alcohol	1
Yeast	*дрожжы / drožžy*	forced to eat	stomach ache in cows (vet)	1

Altogether 93 UI were recorded in general disease categories. The reliability criterion (at least three users) was met by 21 general UI. This group contained 16 different remedies, of which only five were used in two disease categories. The most popular was vodka, used in the respiratory and gastrointestinal categories, followed by goat milk in the general health and respiratory categories, propolis in the general health and respiratory categories, salt in the respiratory and nephrological and urological categories, and goose fat in the respiratory and dermatological categories. Among the remaining 11 remedies, only three were reported by at least five people: pork fat used to treat oral and dental diseases, honey in the case of respiratory diseases and oil in the gastrointestinal category.

The domestic use of non-plant remedies on the individual level was rather restricted, as only one person claimed using spirits in four different illness categories, while two more individuals used honey for treating three different ilnesses.

The informant consensus factor for the whole medicinal use area of other remedies was very low (FIC = 0.67, 175 use citations for 58 remedies). All but three general disease categories had lower FIC values.

**Fig. 3:**
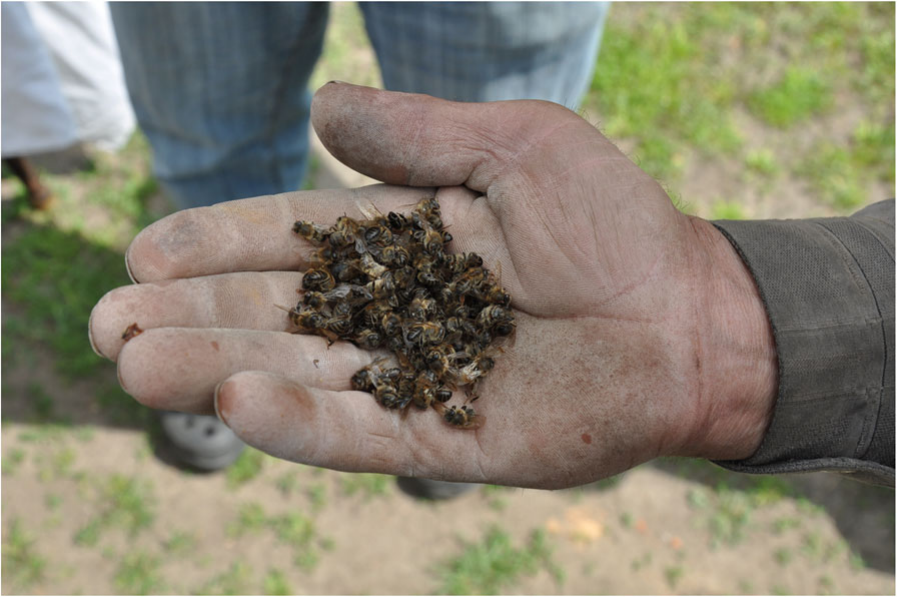
Dead honeybees. Plastok village, photo Raivo Kalle. 13.05.2016

**Fig. 4:**
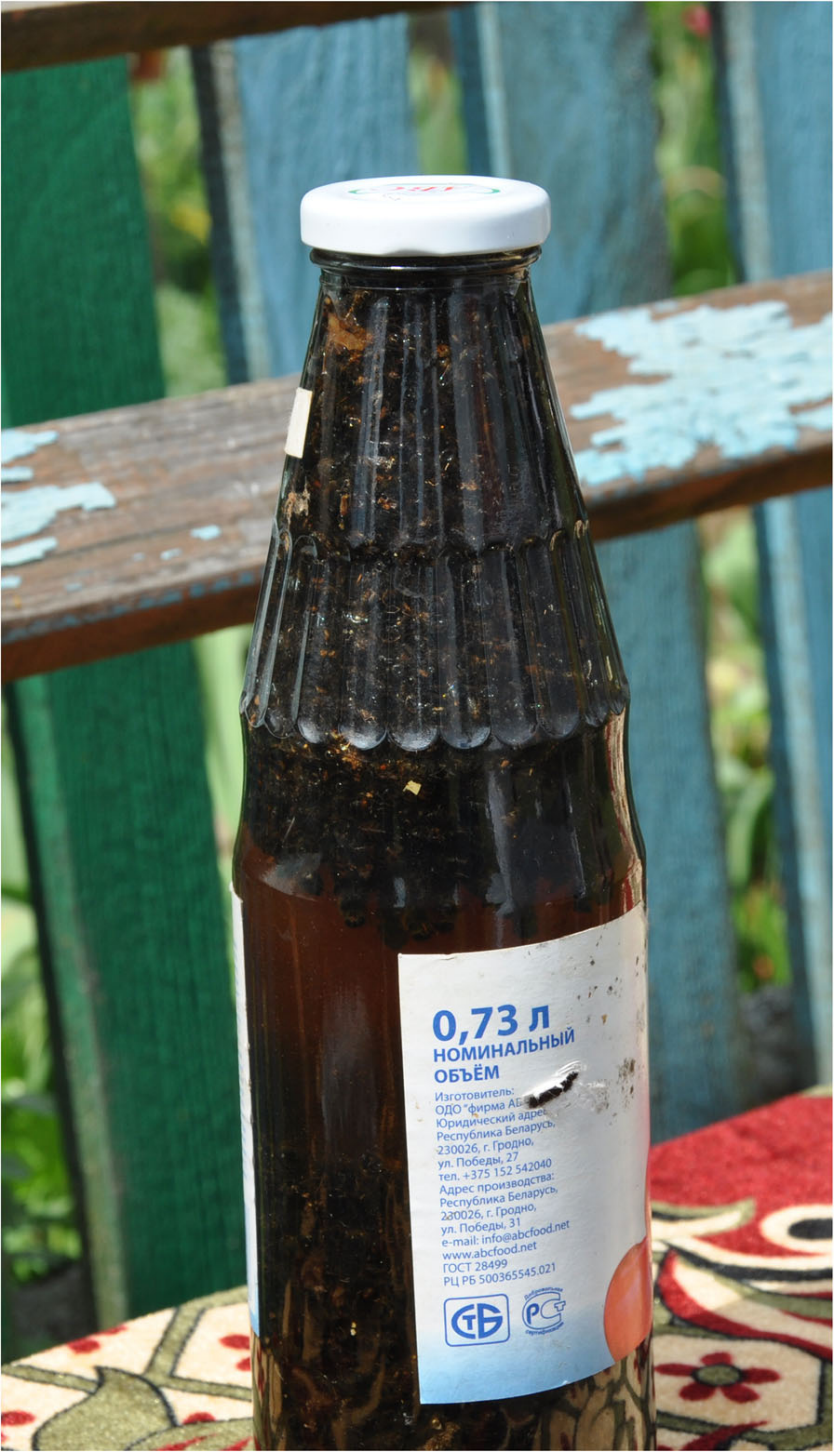
Dead honeybees macerated in moonshine. Plastok village, photo Raivo Kalle. 13.05.2016

The **cardiovascular** category contained only one remedy: dead honeybees (FIC = 1) (Figs. 3, 4). The **oral and dental** disease category had the next highest FIC value (0.85) and contained one dominant remedy (pork fat, either fresh or salted) and two less frequently used ones (topical applications of salt and vodka on an aching tooth). Another small general disease category with a FIC slightly lower than that for the whole use area (0.66) was **nephrological and urological** ailments, containing only two remedies (brandy drunk to treat kidney stones and salted hot water applied externally on cystitis). The **respiratory** disease category was the only extensive one that had a relatively high FIC value (0.68). Among a relatively wide variety of remedies (16), seven met the reliability criterion (having more than three users). Of these the most popular was honey, used mainly to treat cough but also sore throat, and vodka, which was drunk to relieve cold, cough and rhinitis, as well as applied topically to alleviate earache. Goat milk and cow milk with honey and/or soda were used mainly to relieve cough (in children), and heated salt was applied topically to treat sinusitis, sore throat, earache and cough (socks with hot salt). Also, goose fat was mixed with honey and applied or smeared on the chest to alleviate cough and rhinitis.

The **gastrointestinal** disease category (FIC = 0.52, 14 remedies) contained only four remedies that met the reliability criterion. The most popular of these was the use of oil to relieve constipation, vodka to treat stomach ache and diarrhea, kefir to allieviate constipation and starch to cure diarrhea. The **general health** category, containing seven remedies (FIC = 0.53), encompassed only two remedies that met the reliability criterion: the use of goat milk to treat allergies in children as well as to combat cancer, and propolis macerated in alcohol as a panacea. The **infection** disease category (FIC = 0.5, five remedies) contained only one remedy that met the reliability criterion: horse dung water used to treat helminthic infection.

Within the remaining general disease categories FIC values were very low (0–0.3), with only use in the **dermatology** category meeting the reliability criterion: goose fat smeared on burns and foot sores.

A few temporary uses were related to curing particular personal diseases encountered only once in life or during a specific period, such as treating sick children or having access to a remedy (like using fresh milk to treat eye inflammation while keeping a personal cow). Quite a number of recently acquired uses seem to have a traditional origin (e.g. the use of old snakeskins to treat pimples or rolling the soft part of the bread over the stomach, saying a few words and then giving the bread to dogs to alleviate fright), but they could also have been learned from books or journals promoting “traditions”, such as the myth of treating epilepsy with anthills [13]. The popularity of honey and propolis as a medicine is now supported by popular literature (7 uses out of 10 for propolis and 7 out of 8 for honey were attributed to recent acquisition) and may also be due to the massive promotion of apicultural products as good for health [64–66]. Many past uses are spatially distributed among the most popular uses, except a few: burning dog hair to alleviate fright; sheep fat to treat cold and in a salve to alleviate joint pain; lice hidden in bread and given to eat to treat jaundice; hot milk with garlic as a bath to cure helminthic infection; rainwater for washing hair; sweat of horses or sand where chickens bathe to treat warts – all of these, however, were named by only one or two people.

Out of the 134 individuals participating in the study, 60 mentioned the use of other remedies for human medication and of these 23 mentioned only one remedy. The two most knowledgeable individuals recalled 19 and 10 DUR and 17 and 6 used remedies, respectively. The mean number of DUR was 3.3 and for remedies it was 2.7.

#### Comparison with the available historical data

The historical legacy concerning non-plant remedies is much richer than that of cultivated plants which indicates that this remedy group has clearly decreased in number and also in use nomenclature. Historically, symbolic practices of the treatment of diseases were associated with the magical properties of water, fire and stones (soil), and water treatment was widespread [63, 67]. In Belarusian ethnomedicine water was considered a universal remedy in the treatment of almost all diseases, on the basis of both its basic potential to purify the body and the repetitive healing through sanctification, etc. [37]. As common within all traditional medicines around the world, water had, in the opinion of Belarusians, specific powers to heal through bathing (flushing, pouring out). Belarusians treated with water “rosette” (acute pains in the stomach) [43], “evil eye” [43, 63, 68], and a variety of skin diseases [62, 63]; however, such uses could also have been influenced (or earlier local use supported) by widespread hydrotherapy ideas propagated by popular literature, for example the book on water treatment by German doctor Sebastian Kneip (1821–1897). Moreover, the addition of salt [43], metal products [43, 62], coins [43], and ash [69], among other things, was believed to add extra magical powers to water. The first time a child was bathed a coin was thrown into the water, which according to magical beliefs would provide health, skin purity and wealth to the child. One interviewee did recall such a treatment when asked specifically, but her reference was quite impersonal, “second hand” and therefore it is not referred to in the analyzed data, although in other regions of Belarus the use of different metals has been recorded (iron, silver [37], etc.).

Historically fire was an important factor in the process of the symbolic “overbaking” of a sick child in a furnace, a ritual common during the nineteenth century in Hrodna, Minsk and Vicebsk provinces [43, 45, 70]. Likewise, fumigation or smoke was used to cure a fever [39, 69]. Moreover, the historical widespread re-use of ritual objects for fumigation (for example, candles [41, 43, 62] and shells of Easter eggs [62]) was not mentioned by the participants in the current study.

The topical application of stones, widespread historically [45, 62, 63, 71], seems to be largely forgotten, as is the use of all naturally occuring substances apart from salt; for example, minerals (silica [62]), silver (coins [41, 45, 67]) and clay ([41, 45]). The only exceptions noted in this study were the recorded use of sand in which chickens bathed for treating warts, which was clearly indicated as a recalled past use, and the ingestion of clay associated with ethnoveterinary use.

Vodka, however, used historically to dilute some other substances [20, 39], has become a very popular remedy on its own and as a dissolving agent in combination with a variety of plants and, on occasion, non-plant remedies (tinctures for internal or external use).

The use of plant-based products unidentified on the species level, such as the earlier widespread use of tar [41], was only occasionally mentioned. Another plant-based product, bread, was historically attributed protective properties and used in the treatment of various diseases [41, 45, 63, 72]; however, this study recorded the use of bread for treatment only a few times, and in all cases it was used as a mediation substance, conveying words (mumbled on and fed to cows) or as a symbolic means of hiding healing substances (e.g. the remembered past use of hiding lice to treat jaundice). Interestingly, Vallejo and González [73] showed that the use of bread with lice in Spain was encountered in the literature only between 1942 and 1972, although the researched literature extended from 1927 to 2012. The use of lice for the treatment of jaundice was widespread among Belarusians [37]. One recent use (reported as practiced during adulthood, but not during childhood) was the use of bread as a medium for the transfer of fright from humans to dogs. The so-called “out-rolling” with the soft part of bread while still hot was a historically well known magical remedy among Belarusians and other Slavic peoples and used predominantly to remove “fright” from the body. The bread was rolled over the back or stomach and as a result it collected a lot of fur and hair – often used to diagnose the agent “responsible” for the disease. These “out-rolled” diseases were then carried outside human habitats (thrown into water, taken to the forest, left on crossroads) or, more often, fed to dogs. Other substances (such as ashes, wool or boiled eggs) were also sometimes used for the same purpose [74, 75].

Products of animal origin as well as their metabolic products were occasionally used or recalled as having been used in childhood; and all of these were used historically as well. The most common among these included fat [42, 43, 62], apiculture products [63], dairy products [62], and eggs [39, 41, 43, 45, 46, 62, 63, 67, 76], as well as urine [41] and dung water of horse feces and bile [42, 43]. Yet the historical repertorie was much more diverse, containing many products not recalled during our interviews, such as horns and blood [63], feathers [63, 70], placenta [63], waste matter [43, 62, 63], wool and bones [43] and some of the internal organs [63].

The list of the historically used domestic animals and birds is very long, and includes horse [63, 68], pig [45, 63], cow [63], chicken [68], goat [42], cat [63], bull [42], a variety of small mammals (rat [63], bats [43], hare [42], hedgehog [63]), wild birds (cuckoo [42]), reptiles (snake [43, 68], lizard [43]), amphibians (frog [42, 43, 63]), insects (bees [38, 63, 69]) and also fishes (burbot [42], pike [43, 77]). Although it is not always clear how exactly the animal was used in the past, the use of animals often relied on a basic principle - treating similar with similar, with the aim to “transmit” diseases to animals, both by means of direct contact as well as through charms and magical actions (fumigation of wool or feathers of an animal/bird that caused the consternation [63, 69]). Among the records of the present study, mainly practical and seemingly quite rational uses of animal (products) have continued to be used.

Among the human-originated substances, the historical data list such remedies as human placenta [67], hair [38, 41, 63], nails [41, 45, 63], and the urine of sick people [20, 43]. Items bearing symbolism of the underworld include, for example, objects belonging to the deceased or a cemetery [43, 45, 62], spider’s web [43, 45], moss (from the roof of the house) [43, 77] and a piece of cloth with menstrual blood [77]. None of these were mentioned during the interviews in this study.

#### Charm-healing in the context of lay-treatment

The recording of charms was not the purpose of the present study as healing with charms and healing with plants are usually perceived as separate categories of healing, considered independent. Yet in a few cases we encountered some elements of healing concerned with the use of charms that are related to the use of non-plant remedies, such as alleviating fright by rolling bread over the stomach. The use of plants both in magical practices and as accompainments to charms is based not only on their real biological characteristics, but also on the laws of mythological logic - the place of the plant in the traditional worldview [78]. The use of plants in folk medicine is often based on the principle of signatura rerum, when people believe that the plant itself (by its color, shape, etc.) indicates which disease they need to treat. In magical practice in general, the correlation of the external manifestations of a disease, especially the cutaneous ones, with a particular plant is important. This is particularly evident in the example of the ritual removal of warts, for which comparably round potatoes, peas and apples were used: “People took potatoes and rubbed them on the warts. Potatoes cut in half, applied on the warts, and then pressed together and buried. As soon as the potato dries the warts should disappear” [37]. All across the country this practice continues to use prickly, scalding or even poisonous plants as apotropes, for fumigating a patient with the magical intention of expelling the spirit of the disease. For example, children that had received a fright were fumigated with agrimony, among other things. Other features of the plant have become more relevant, such as the multiplicity of poppy and flax seeds, when sprinkled over a child afflicted by the evil eye, etc.

Treatment with words, which was embodied in the practice of medical charms, was historically considered an important way to get rid of disease along with remedies of plant, animal and mineral origin. Charms were mainly used as prophylactics and to treat children’s and women’s diseases, infectious diseases, skin diseases, neuropsychiatric conditions, dental issues and complaints related to the digestive system, head, eyes, ears, nose and throat, as well as fear, the evil eye, and health problems related to childbirth. This type of treatment was conducted by healers, although some proportion of the population had basic folk medical knowledge. The charms are accompanied by certain actions which reinforce the magical meaning of the act of treating.

Earlier folklore collectors noticed that "treatments with charms exist everywhere and belief in them was unusually high" [20]. During fieldwork for this study many interviewees mentioned as a past phenomenon whispering old women (бабка-шаптуха, babka-šaptucha) who were consulted in the course of specific diseases (such as fright and evil eye). However, a few lay interviewees also recited some charms. One of the villages even had their own healer man, who was still practicing healing with charms. Today the practice of applying to healers is reserved for illnesses of the psycho-neurological sphere (fright, evil eye, insomnia) and certain skin diseases (for example, erysipelas).

### Veterinary medication

#### Cultivated plants

There were 48 DUR referring to the use of 14 taxa from 12 families, of which only two families, Asteraceae and Cucurbitaceae, were represented by two taxa. The most commonly used taxa, and the only one named by 14 people, was *Linum usitatissimum* (18 DUR). Three additional taxa met the reliability criterion: *Solanum tuberosum*, *Cucumis sativus* and *Anethum graveolens* (three users and DUR for each). The diversity of uses on the individual level was very low: one person used two plants and five people used one plant for treating two different animal illnesses.

The informant consensus factor for the whole veterinary use area of cultivated plants was relatively low, yet similar to the FIC of wild plants (FIC = 0.69, 43 use citations for 14 taxa). The only individual general category with the same FIC value was the emic category **rumination problems**, for which six taxa were used; however, only one of them, *Linum usitatissimum,* met the reliability criterion (eight users). All other disease categories had low FIC values within the range of 0.2–0.56. The only two uses that met the reliability criterion within those categories consisted of *Linum usitatissimum* to treat diarrhea in both cows (seven users) and pigs (five users).

There were no extraordinary uses of cultivated plants that have been attributed only to past or present contexts, except for the recent adoption of the feeding seeds of *Piper nigrum* to chickens in order to strengthen them. The taxa *Linum usitatisiiumum* was temporally dynamic: two uses were claimed to have been acquired during adulthood (rumination problems and diarrhea in cows) and seven uses were attributed to the past, of which six were divided equally among diarrhea in cows, diarrhea in pigs and rumination problems in cows; however, the same uses were also continuously utililized.

Out of the 134 individuals participating in the study, 26 mentioned the use of cultivated plants for veterinary purposes. The most knowledgeable person mentioned four taxa (and six DUR), whereas 18 people mentioned only one taxon. The mean number of used cultivated taxa was 1.4 while the mean DUR for this group was 1.7.

##### Comparison with the available historical data

Most popular or widespread cultivated plants noted in nineteenth century literature appear to be no longer used. Some of them (such as *Nicotiana* spp. which was indicated for the treatment of cow, horse and sheep diseases [48, 49, 52]; and *Cannabis* *sativa* [48–50] which was used to treat dogs, horses and sheep) have been officially banned for domesti cultivation in different periods of 20th century, whereas others (such as some cereals (*Secale cereale, Hordeum vulgare, Avena sativa, Fagopyrum esculentum*) [48] used for cow, horse and pig healing) simply stopped being grown in home-gardens due to fundamental changes in the economic sphere occurring in the twentieth century. Other historically known cultivated plants include garlic which was used for the treatment of wounds (especially on the tongue of ruminants) as well as against sheep parasites (*матыліцы*) [48, 51, 52] and various vegetables (e.g. radish [49], horseradish, cabbage, carrot [48]) used to treat internal diseases of cows and horses. None of these was used by our interviewees.

#### Non-plant remedies

In ethnoveterinary medicine, 72 DUR referring to the use of 31 non-plant remedies were identified. The most commonly used remedies were vodka (13 DUR) and laundry soap (eight DUR). Twenty remedies were used by one person only. Five additonal remedies met the reliability criterion: urine (five users), egg and vodka, beer, horse feces and clay (three users each). The diversity of uses was almost as low as for cultivated plants: one person used vodka for three different emic animal illnesses, while six more people used various remedies for treating two different emic animal illnesses.

The informant consensus factor for the whole veterinary use area of non-plant remedies was higher than for the human medication use area of non-plant remedies and similar to the FIC of cultivated and wild plants (FIC = 0.57, 70 use citations for 31 remedies). Only two of the individual general categories had FIC values higher than that for the whole use area.

**Mastitis** was the disease category with the highest FIC value (0.71); and in this category five different remedies were used, two of which, laundry soap and urine, met the reliability criterion. The category of **helminthic infection** had a FIC value of 0.66 and contained two remedies, of which the use of horse feces met the reliability criterion. All other disease categories had low FIC values within the range 0.18–0.5. Within these only a few uses met the reliability criterion: vodka and beer were force-fed to cows to treat rumination problems, a mixture of eggs and vodka was given to piglets to cure diarrhea and laundry soap was given to cows to alleviate constipation.

Over 40% of the DUR were described as used in the past; the majority of them were among the most widely used remedies, while some were uniquely used remedies, such as pouring salted water into the perforated side of a calf or giving soda to goats to drink to treat rumination problems, and the smearing of pigs with tar to cure red fever or scabs. Over 60% of the uses of vodka were also attributed to the past (when animals were still kept by many people). None of the uses in this remedy category were reported as recently acquired.

Out of the 134 individuals interviewed, 30 mentioned the use of non-plant remedies for veterinary purposes, and of these people 12 mentioned only one remedy. The most knowledgeable person mentioned eight remedies, and two other individuals mentioned five remedies and DUR. The mean number of non-plant remedies was 2.3 while the mean DUR of this group was 2.4.

##### Comparison with the available historical data

Significant quantitative predominance of non-plant remedies over cultivated plants used for treating domestic animals seems also to be historically specific for Belarusian ethnoveterinary medicine. Some of these remedies were still in use or recalled as past uses. The universal non-plant remedies (stones [48, 52], water [48, 49, 53], fire [48, 50, 54], bread [48, 50, 52], salt [48, 50] etc.), which have already been discussed above in the context of ethnomedicine, were also widely used in veterinary medicine for both treating and preventing animal diseases (*mastitis*, rabies in dogs, snakebites, digestive disorders of cows and horses, cow placental retention (*retentio placentae*), skin diseases of cattle and horses, “the evil eye”, etc.). The most numerous and diverse group of non-plant remedies consisted of animal-based ones, such as different kinds of animals and parts of their bodies (insects [48, 49, 52, 53, 55], birds [49], mammals [48, 49, 52, 53], fishes [48, 52], amphibians [48, 48]] and reptiles [48]) or substances obtained from human and animal bodies (fat [49, 52], urine [48, 50, 52], feces [48, 49, 51, 52], placenta [49, 52]], bile [48, 49], blood [49, 52]) that were used to treat ungulates (horses, cows, sheep and pigs). Honey [48–51, 54] and the application of birch tar [48, 49, 56] played an important role in treating infectious diseases of horses, cows and pigs. Various milk products were used to treat ungulate skin diseases [49, 51], as well as urination [49] and digestive [48, 50] problems in cattle. Eggs were considered to be a universal remedy for cow and horse diseases [49, 52].

##### Comparison between the use of wild and cultivated plants and non-plant remedies

While modern complimentary and alternative medicine has been perceived as a self-driven choice and possibility for caring for one’s self [79], the “remains” of traditional healing practices have a kind of ambivalent position, valued as traditional relicts, yet often perceived as something less effective, and even shameful (for example, the use of urine, and even the use of vodka). As many (especially wild) plants used in ethnomedicine belonged to the official repertoire of Soviet medicine, it seems that this group of home remedies had been silently given “use consent” in home-healing.

**Fig. 5:**
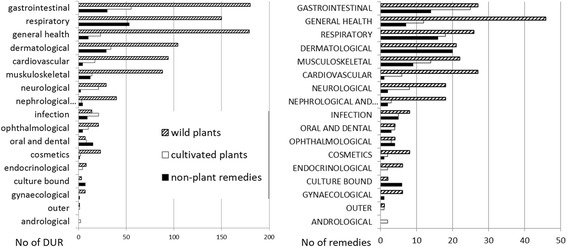
Division of the number of DURs and used remedies within three remedy groups and different human disease categories; on both sides of the graph disease categories are listed according to the sum of all DURs or used remedies in the category

All human disease categories have been predominantly treated with wild plants and only two categories, namely culture bound and oral and dental, had more extensive use of non-plant remedies, although the nomenclature of the remedies for dental treatment was rather limited (Fig. 5, Table 3).

**Table 3:** Distribution of emic disease categories among general disease categories and different domains. UCcp – Use Citation of cultivated platns, UCnonp –Use Citation of nonplant remedies, UCwp –Use Citation of wild plants. ΣUT/R – sum of used plant taxa / other remedies; FIC – Informant Consensus Factor

Emic disease categories / General disease category – ΣUT/R (FIC)	UCcp	UCnonp	UCwp
Andrological	2 (0)	0	0
Men’s diseases	1		
Potency	1		
Cardiovascular	6 (0.64)	1 (1)	27 (0.62)
Anemia			1
Bad blood			1
Haemostatic			4
Heart diseases			9
Heart problems	9	2	34
Hypertension	7	2	34
Hypotension	1		3
Promotes bloodstream			2
Thickening of blood			1
Varix			4
Vesical cleaning			1
Cosmetics	2 (0)	1 (0)	8 (0.67)
Rejuvenating			2
Beauty procedure	1		1
Hair care	1	1	19
Dandruff			1
Culture bound	0	6 (0.17)	2 (0.5)
Against a fright from lightening		1	
Evil eye		1	1
Fright		5	1
For women to be strong against men			1
Dermatological	10 (0.69)	20 (0.3)	21 (0.81)
Abscesses	3		4
Allergies			1
Burns	11	9	7
Cuts	2	1	7
Eczema		2	2
Foot sores		3	3
Inflammation	1		1
Inflammation after injection	1		
Pimples		1	
Psoriasis		1	
Rotten wounds	2		1
Scabies			1
Skin diseases	4		4
Splinter in the finger		1	
Tumor			1
Warts		8	10
Wounds	10	3	62
Endocrinological	2 (0.5)	0	6 (0.29)
Diabetes	2		5
Hyperthyroidism	2		2
Pancreas			1
Gastrointestinal	25 (0.59)	14 (0.52)	27 (0.78)
Bile neutralizer			1
Bile deficiency			1
Cleaning of stomach		1	
Constipation	7	10	4
Gastritis			4
Gastric ulcer			7
Gall stones			1
Dysentery			4
Diarrhea	20	12	63
Flatulence (in children)	2		
Hemorrhoids	4		1
Jaundice		1	
Laxative	3		
Liver diseases	4		17
Low acidity			1
Stomach problems	1		7
Stomach ache	14	5	69
Vomiting		1	
General health	12 (0.5)	7 (0.54)	46 (0.75)
Allergies			2
Appetizer			3
Aroma therapy			1
Body cleansing			5
Bone strengthening			1
Cancer	2	2	3
CO-intoxication			2
Diathesis in children	1		12
Disinfectant			3
Fever		1	3
Good for health			6
Hangover			1
Wellbeing	7		49
Immune boosting	4		7
Inflammation processes			5
Men’s health	1		
Organism cleansing			7
Pain			1
Panacea	5	7	32
Prophylactics	1		26
Strengthening of organism	2		4
Tonus support			1
Washing hair			1
Vitamins			4
Gynecological	0	1 (0)	6 (0.17)
Women’s diseases		1	6
To increase human milk production			1
Infection	5 (0.79)	5 (0.5)	8 (0.46)
Anti-microbic			1
Chickenpox			1
Helminthic infection	20	6	9
Scabies			1
Tuberculosis	1	3	2
Musculoskeletal	14 (0.54)	9 (0.2)	22 (0.74)
Back pain		1	7
Bruises			3
Foot ache		1	8
For adhesion of bones			1
Joint pain	12	9	50
Knee ache			2
Rheumatic pains	2	1	17
Nephrological and urological	3 (0)	2 (0.67)	18 (0.54)
Cystitis		3	
Diuretic	1		7
Kidney diseases	1		29
Kidney stones	1	1	1
Urinary bladder problems			2
Urinating problems			1
Neurological	8 (0.59)	2 (0)	18 (0.35)
Calming			6
Convulses			1
Epilepsy		1	4
Headache	8	1	11
Insomnia			1
Nerves			2
Sedative	12		1
Soporific	1		3
Ophthalmological	3 (0.78)	4 (0)	4 (0.84)
Eye inflammation	2	1	
Eye pain	2	2	
Eye problems	6	1	16
Improve vision			5
Oral and dental	4 (0.57)	3 (0.85)	4 (0.5)
Gingival inflammation	1		
Gingival bleeding			2
Gingival diseases			1
Gingival wound			1
Periodontitis			1
Teething pain in children			1
Toothache	7	15	1
Other	1 (0)	0	1 (1)
Antitoxic			1
Snake bites	1		
Respiratory	18 (0.63)	16 (0.68)	26 (0.84)
Asthma		1	
Bronchitis			6
Cold	19	7	94
Cough	8	23	42
Earache	3	5	1
Expectorant			1
Lung diseases	4		2
Pneumonia			2
Rhinitis	13	2	2
Sinusitis		2	
Sore throat	4	13	

**Fig. 6:**
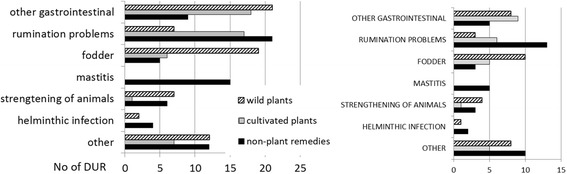
Division of the numbers of DURs and used remedies within three remedy groups and different ethnoveterinary categories; on both sides of the graph disease categories are listed according to the sum of all DURs or used remedies in the category

In ethnoveterinary medicine, non-plant remedies have been relatively more important than both wild and cultivated plant remedy groups. However, only one ethnoveterinary category, namely mastitis, has been treated solely with non-plant remedies, while another (rumination problems) boasts both the largest number of uses and the greatest diversity within the non-plant remedies (Table 4, Fig. 6). Cultivated plants have had modest application in ethnoveterinary medicine, slightly more prominent in the diversity of taxa used only in the category of gastrointestinal problems other than rumination.

**Table 4:** Distribution of emic ethnoveterinary categories among general ethnoveterinary categories and different means of treatment. UCcp – Use Citation of cultivated plants, UCnonp –Use Citation of non-plant remedies, UCwp –Use Citation of wild plants. ΣUT/R – sum of used plant taxa / other remedies; FIC – Informant Consensus Factor

Emic etnoveterinary categories / General ethnoveterinary category – ΣUT/R (FIC)	UCcp	UCothr	UCwp
Gastrointestinal	9 (0.56)	5 (0.5)	8 (0.63)
Constipation in cows	1	3	
Diarrhea in calves	1	2	2
Diarrhea in chickens			1
Diarrhea in cows	9		10
Diarrhea in piglets		3	
Diarrhea in pigs	7	1	8
Fodder	5 (0.2)	3 (0.33)	10 (0.55)
Fodder for bees in spring			1
Fodder for cows			3
Fodder for home animals			2
Fodder for pigs	2		5
Fodder for rabbits			4
Fodder for turkeys			3
Fodder to increase milk production in cows	4	3	1
Fodder to make egg-shells stronger		2	
Helminthic infection	0	2 (0.67)	1 (0)
Helminthic infection in pigs		3	1
Helminthic infection in dogs		1	
Helminthic infection in cows			1
Mastitis	0	5 (0.71)	0
Mastitis in cows		15	
Rumination problems	6 (0.69)	13 (0.4)	3 (0.67)
Rumination problems in calves		1	
Rumination problems in cow	17	11	6
Rumination problems in goats		1	1
Stomach ache in cows		4	
Stomach problems in cows		4	
Strengthening of animals	1 (0)	3 (0.33)	4 (0.5)
Given to claves		2	
Goat kids’ illness		1	
Strengthening of cows			1
Strengthening of piglets			1
Strengthening of pigs		1	4
Vitamins for cows			1
Vitamins for claves		2	
Weakness in chickens	1		
Other	5 (0.43)	10 (0.18)	8 (0.3)
Appetizer for domestic animals	1		1
Appetizer for cows			2
Bacterial diseases in bees			1
Blood in urine in cows			1
Cold in domestic animals			1
Cuts in domestic animals			1
Disinfection for home animals			1
Dog plague		2	
Eczema		1	
Eye inflammation in cats	1		
Eye pain		1	
Good for cows			1
Good for domestic animals	1		
Good for horses			1
Good for pigs	4		
Red fever in pigs		1	
Scabs		1	
Skin diseases in pigs and piglets		1	
Skin diseases in pigs		1	
To protect cows		1	
To protect cows against snake bites		1	
When an overheated horse has drunk cold water		1	
When piglets do not go to their mother			1
Wounds in cows			1
Wounds in horses		1

**Fig. 7:**
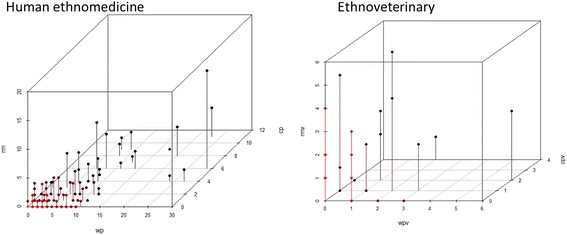
Comparison of the numbers of non-plant remedies (rm(v)), wild plants (wp(v)) and cultivated plants (cp(v)) used by each person who claimed human ethnomedicinal or veterinary use within any of the remedy groups. Colored dots represent more than one interviewee

The entire diversity in the use of both ethnomedicinal and ethnoveterinary remedies was created by only a limited number of people, providing many use records (Fig. 7). Even though there were a remarkable number of people (41) who named only wild plants used in human ethnomedicine, there were also six people who named only cultivated plants and/or non-plant remedies. The situation for ethnoveterinary medicine differs slightly as a similar proportion of individuals used only non-plant remedies (eight people), cultivated plants (seven people) or both (six people), and a relatively smaller proportion (compared to human medicine) of people (14 individuals) used only wild plants to attend animals. The occurrence of people in the sample who have used only non-plant remedies indicates that, even if asking about wild food plants first may have predisposed some respondents to concentrate on wild plants, it was not universal.

**Fig. 8:**
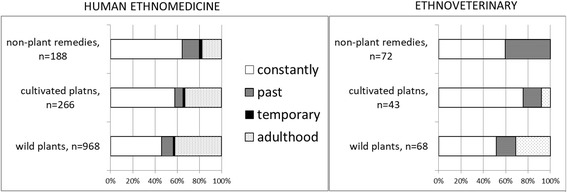
Proportion of DUR in different temporal blocks in human ethnomedicine and ethnoveterinary medicine

In human ethnomedicine wild plants constitute the most changeable group of remedies, with over 40% of uses adopted later in the life (Fig. 8). Other remedies contribute the highest proportion of continuous uses as well as the highest proportion of the past uses, while recently acquired uses represent the lowest proportion. Cultivated plants fall in between the other two remedy groups, contributing a very small proportion of past uses, while recent additions seem to be quite numerous, but still representing a smaller proportion than among wild plants. Among the remedy groups in the ethnoveterinary category the difference regarding the recently acquired uses is noteworthy: almost 30% of the uses of wild plants in contrast to none for non-plant remedies. The non-plant remedy group also boasts the greatest proportion (over 40%) of uses attributed to the past only. None of the uses in the ethnoeterinary use area were referred to as temporary.

Comparison of the overlap of remedies in the human medicinal and ethnoveterinary use areas shows an interesting anomaly in the non-plant remedies category. A vast majority (all but three taxa) of the wild plants and all but one cultivated plant used in ethnoveterinary medicine were also used for human medication. Among non-plant remedies the overlap is considerably less: 14 remedies out of 31 used for ethnoveterinary medicine did not have any other use in human medicine.

Therefore, non-plant remedies can be perceived as the remnants of traditional healing which is no longer evolving. As this group is not supported by official medicine, it has less new uses (acquired during adulthood) and more past uses, and the group may continue to diminish, as has already happened compared to the widespread historical use in Belarus. The more extensive use of non-plant remedies in the ethnoveterinary use area also supports this idea, as ethnoveterinary medicine in Belarus seems to be of marginal importance due to well-developed and accessible professional veterinary care and thus it is practiced by few enthusiasts only.

##### What supports sustainability of wild plants in human medicine and non-plant remedies in ethnoveterinary medicine?

Wild plants are more versatile because of their availability, diversity and sustainability with regard to the local climate. A significant number of uses of wild plants are associated with knowledge obtained from the media (such as *Narodnaya gazeta* [59], *7 dnej* [60]) as well as from the older generation. For example, publications have promoted the use of twigs of trees and berry bushes for tea [61], berries for compresses, rinses and baths [62], and inflorescences for decoctions and ointments [63].

About 33% of the study area is covered by forests, 27.5% of which are man-made, mainly consisting of coniferous plantations. Coniferous, birch, and black-alder forests, as well as oak and fir trees can be encountered. In the mid-1920s, a large land reclamation initiative began in the territory of the district. The peak of melioration fell on 1960–1970s [64]. In total, more than 70,000 ha of swamps were drained in the Liubań region alone. As a result of land reclamation, the area of agricultural land increased significantly (the total area of agricultural land is now 79.2 thousand hectares, of which 62.3 thousand ha are drained); however, due to a number of mistakes and miscalculations, some parts of this land began to be covered with sand dunes, on which nothing grew. Today, marshes cover 0.7% of the Liubań region [14].

In the territory of the Liubań district there are 3 hospitals, a polyclinic, 10 dispensaries, 14 first aid points, and 6 pharmacies. As a rule, pharmacies are located in towns. In villages, medicines can only be purchased at first aid stations [14]. Veterinary help is provided by veterinarians who work in collective farms, as well as specialists who are retired [80]. In spite of the well-organized professional assistance available, folk veterinary medicine, or a set of methods based on both natural science knowledge about animals and the traditional mythopoetic model of the world, continues to actively adhere to folk remedies, or the use of vegetable, animal, mineralogical, and magical medicinal products, including charms. The latter decreased, in comparison with the post-war period, by the 1980s (the time of active collection of material in the Paliessie region). This was due to urbanization and the decline of rural households as well as ideological changes in society, which brought about the decay of traditional culture. At the same time a vast store of traditional knowledge about animal treatment is preserved in the passive memory of rural residents. Also, their use may be encouraged by veterinarians, a significant portion of who are of rural origin themselves and thus they use “grandmother’s” remedies in their professional activities. Non-plant remedies may also still be important in veterinary practice due to the influence of popular literature (magazines, newspapers) and media.

Knowledge and skills from the field of folk medicine, apart from the use of plant and animal derived products, tend to be in the realm of the sacred. In regard to Soviet atheism they were painted in a negative light with the label “superstition” and pushed underground. This may also be the reason why the respondants in this study did not report them during the interviews, especially as the conversation started with plants and no leading questions were subsequently asked. Conducted interviews did not presume discussions about the healing properties of water, metal, fire or stones. The lack of comments pertaining to a number of folk magic rituals does not at all indicate their absence in the passive memory of the rural residents of the Liubań region or maybe even in their practical use; rather, it is not regarded as the type of knowledge to be willingly shared with a stranger, as is the use of plants for healing. As the use of non-plant remedies was quite recently considered superstition and the “remains of old times”, not suitable for the new social reality, it may well be that people are not willing to open up and speak freely so as not to sound unknowledgable. However, as non-plant uses are quite numerous for the ethnoveterinary use area, and some people reported only the use of non-plant remedies in either human or veterinary medicine, this assumption may not be entirely accurate.

## Conclusions

This study examined the use of cultivated plants and non-plant remedies in human and ethnoveterinary mecidine and also tried to understand the importance of these groups of remedies compared with wild plants. While there may be a little bias in favor of wild plants due to the way information was collected (starting point of the interview was wild food plants), the differences among the three remedy groups were well pronounced, indicating that in domestic medicine cultivated plants and other means were remarkably less important than wild ones. In ethnoveterinary medicine non-plant remedies were nearly equally important as wild plants, while cultivated plants were the least utilized. However, being “at hand” does not necessarily mean being used or valued more, while remedies requiring more specific knowledge (such as wild-growing plants) are used more often and diversely. Even against the backdrop of the loss of unintentional contact with nature, people in Belarus more readily talk about using wild plants, supported by both official medicine and popular literature. While the use of non-plant remedies is somewhat promoted by popular literature, this alone does not seem to be sufficient to sustain local uses and/or open people up for sharing such uses. Therefore, even if our results indicate that proximity to the domestic arena does not influence the popularity of a remedy, we cannot say this with full certainty. Further research should develop more structured and detailed approaches and methods for evaluating the trends detected in the present study in order to better understand the mechanisms of the evolution of popular medicine.

## Abbreviations

DUR: Detailed Use Records
FIC: Informant Consensus Factor
UC: Use Citations
UI: Use Instances
